# Experimental Investigation of the Phase Relations in the Fe-Zr-Y Ternary System

**DOI:** 10.3390/ma15020593

**Published:** 2022-01-13

**Authors:** Chenbo Li, Qian Song, Xianwen Yang, Yuduo Wei, Qi Hu, Libin Liu, Ligang Zhang

**Affiliations:** School of Material Science and Engineering, Central South University, Changsha 410083, China; 193112081@csu.edu.cn (C.L.); 203111046@csu.edu.cn (Q.S.); 213112124@csu.edu.cn (X.Y.); ol1fs5bov1e@163.com (Y.W.); 203112137@csu.edu.cn (Q.H.); pdc@csu.edu.cn (L.L.)

**Keywords:** Fe-Zr-Y system, phase equilibria, solid solubility, isothermal section

## Abstract

The phase relations of the Fe-Zr-Y system at 973 K and 1073 K were experimentally investigated by using the equilibrated alloys. New ternary compounds τ3-Fe_3_ZrY and τ4-Fe_10_Zr_5_Y_2_ were found in this ternary system. The solubility of Y in Fe_2_Zr was measured to be 3.5 at.% and the third component can hardly dissolve in the other binary intermetallic phases. Experiments have verified that Fe_2.9_Zr_0.5_Y_0.5_ has a solid solubility ranging from Fe73Zr12Y14 to Fe77Zr9Y13.

## 1. Introduction

Due to the use of nuclear fusion and the third-generation nuclear fission reactors, zirconium-based alloys have been widely studied as important nuclear cladding materials [1,2,3,4,5]. This relies on their specific characteristics: excellent corrosion resistance, good mechanical properties, high resistance to radiation damage and a low cross section of capture for thermal neutrons [6]. The Fe-Zr-Y system is an important member of the zirconium-based nuclear cladding materials, the high temperature resistance and radiation resistance of which can be greatly improved after oxidation [7,8,9,10,11]. Although the Fe-Zr-Y system has excellent prospects, there are still some problems that limit its applications in industry. For example, when the temperature of a nuclear reactor core continuously rises, the Fe-Zr-Y system may be at risk of being unstable [12].

An efficient and effective solution to this problem is to obtain a comprehensive and profound understanding on phases of different components and rule out the phases that are unstable at high temperatures in advance. which is of vital importance for the applications of the Fe-Zr-Y system [13]. The purpose of this work is to explore the phase diagram of the Fe-Zr-Y system at high temperature in order to intuitively express the relationship between phases under the thermodynamic equilibrium state so as to provide a basic theoretical guide for the research, development, and design of new materials.

**Figure 1:**
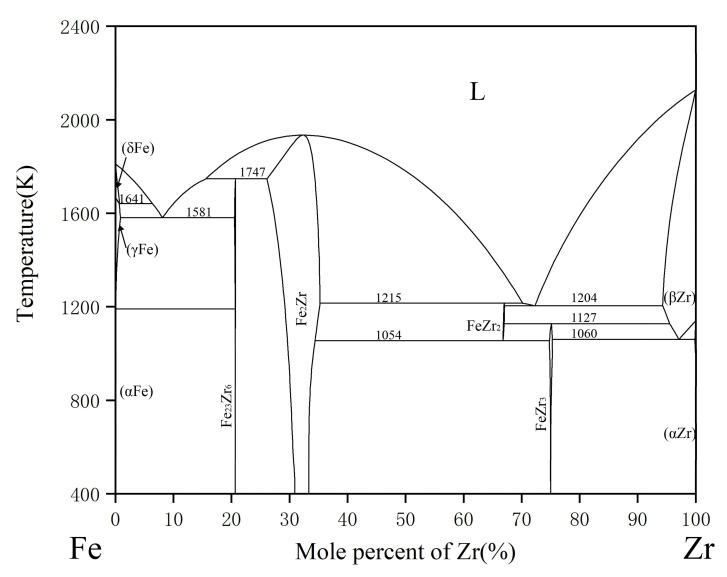
The calculated Fe-Zr phase diagram based on the work of Lu et al. [30].

The experimental investigations on the phase relations of the Fe-Zr-Y system [14,15,16,17,18,19,20] have been carried out, and several assessments of thermodynamic data have been obtained [21,22,23,24,25,26]. Although there are some controversies about the stability of hex-Fe_2_Zr and Fe_23_Zr_6_, it is widely accepted that both of the two phases exist [3]. According to the results of X-ray and magnetic measurements, Kai et al. [27] confirmed the presence of hexagonal Fe_2_Zr, which had the same structure of MgNi_2_. Subsequently, Stein et al. [28] found the Fe_2_Zr phase of C36-type structure through heat treatment at 1563 K. Meanwhile, Liu et al. [16] confirmed the existence of Fe_23_Zr_6_ by using a transmission electron microscope (TEM) and a scanning transmission electron microscope (STEM). Recently, Yang et al. [29] and Lu et al. [30] applied first-principles calculations to evaluate the formation enthalpy of compounds in Fe-Zr system, in which Fe_23_Zr_6_ was considered as a stable phase. After a thorough survey and thermodynamic evaluations based on a number of experiments, the binary phase diagrams reported by Lu et al. [30] were finally adopted in this work. As shown in Figure 1, among the four intermetallic phases, only Fe_2_Zr phase has an obvious homogeneity range. In other phases, FeZr_2_ appears in the temperature range of 1215–1054 K, and FeZr_3_ is formed through peritectoid reaction.

**Figure 2:**
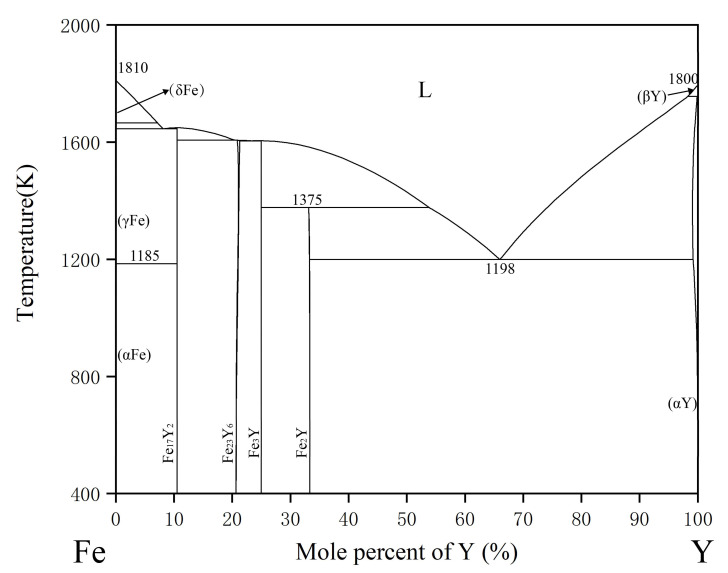
The calculated Fe-Y phase diagram based on the work of Saenko et al. [34].

Domagala et al. [31] reported the Fe-Y phase diagram for the first time in the whole composition range. Then, a thermodynamic assessment was carried out by Gschneider et al. [32] Recently, Zhang et al. studied the crystallography and thermodynamics of the compounds in the system, which provided a basis for further optimization of the Fe-Y system [33]. Figure 2 shows the Fe-Y phase diagram with four intermetallic compounds: Fe_17_Y_2_, Fe_23_Y_6_, Fe_3_Y and Fe_2_Y. Based on the previous research work mentioned above, Saenko et al. [34] reported the thermodynamic optimization of the Fe-Y binary system, as shown in Figure 2.

**Figure 3:**
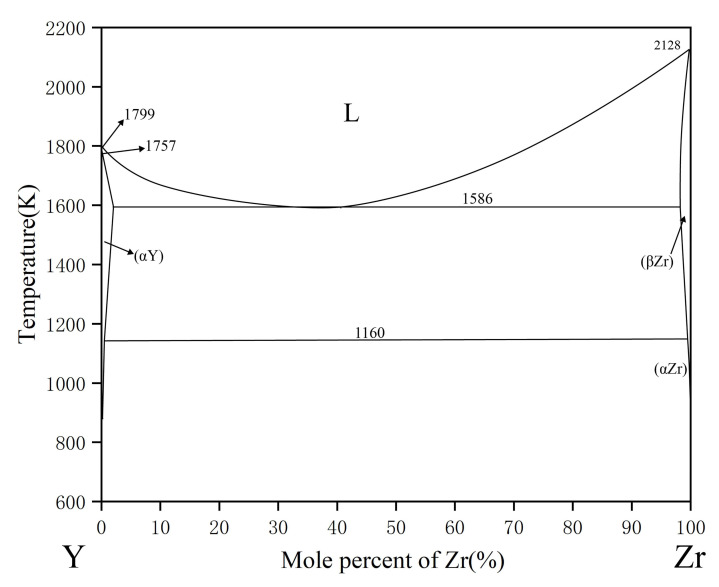
The calculated Y-Zr phase diagram based on the work of Bu et al. [38].

The phase equilibrium relation of Zr-Y phase diagram was first studied by Wang [35], based on which Palenzona and Ciraflci [36] optimized the thermodynamic data. Recently, Flandorfer et al. [37] constructed the thermodynamic database of Zr-Y system. The calculated phase diagram used in this work adopts the latest thermodynamic parameters reported by Bu et al. [38], as shown in Figure 3.

**Figure 4:**
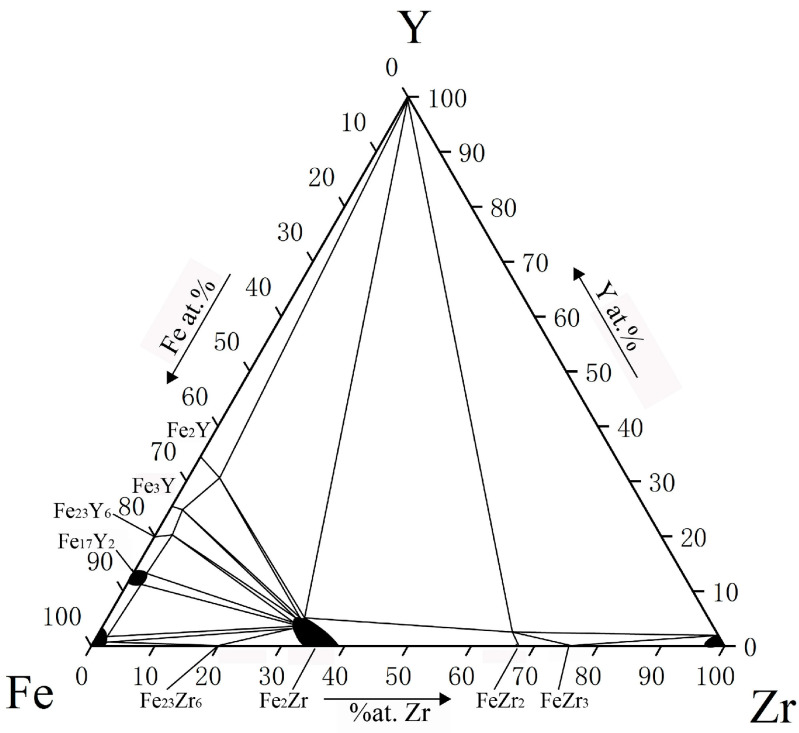
The 1070K isothermal section of the Fe-Zr-Y ternary system [39].

Research on the phase relationships and ternary compounds of Fe-Zr-Y ternary systems was relatively limited. In 1986, by combining three related binary phase diagrams, Harchenko et al. [39] measured the 1070K isothermal section of the Fe-Zr-Y ternary system for the first time, but no ternary compounds were discovered, as shown in Figure 4. In 1989, Jifan et al. reported Fe_9_Zr_2_Y phase with ThMn_12_ structure [40]. At the same time, Itoh et al. also found Fe_2.9_Zr_0.5_Y_0.5_ phase of Orthorhombic type [41]. The binary and ternary phases, the crystal structure and lattice parameters that were previously reported are listed in Table 1.

## 2. Experimental Procedures

The phase relationship of the Fe-Zr-Y system was studied through the equilibrium alloy method of static measurements. Iron rod (99.99 wt.%), zirconium rod (99.99 wt.%) and yttrium block (99.99 wt.%) were selected as raw materials. The weight of the samples was controlled around 12 g, with an error of ±0.005 g. The samples were placed in a non-expendable arc melting furnace with high purity argon atmosphere. At the same time, a sponge titanium button was added as the oxygen absorber to prevent oxidation. Each alloy button was melted for at least 4 times to ensure its uniformity. The alloy buttons were divided into two parts and sealed in the quartz tubes filled with argon. According to the reported heat treatment processes of similar systems [3], 60 and 90 days were set as the annealing times for 700 °C and 800 °C, respectively. After annealing, the samples were quenched in cold water to preserve the microstructure at high temperature.

The morphology and phase compositions of the alloy were analyzed by electron probe microanalysis (EPMA, JAXA-8800R, JEOL, 15 kV, 1 × 10^−8^ A, Tokyo, Japan). X-ray diffraction was employed (XRD, Rigaku d-max/2550 VB, Cu K, 40 kV, 250 mA, Tokyo, Japan) to analyze the crystal structure of typical alloys, with the scanning range of 10–90° and a speed of 0.133°/s. Backscattering electron (BSE) images of the alloy samples were acquired using a scanning electron microscope (SEM, TESCAN MIRA3 LMH, 15 kV, working distance of 15 mm, Brno, The Czech Republic).

## 3. Experimental Results

**Figure 5:**
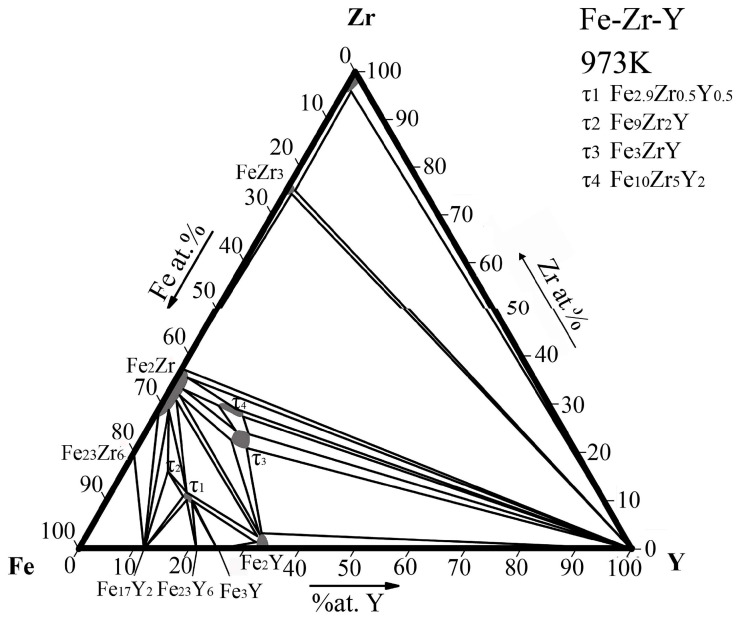
Isothermal section of the Fe-Zr-Y ternary system at 973 K determined in this work.

Based on the phase equilibrium data of 24 alloy samples, the isothermal section of the Fe-Zr-Y ternary system at 973 K was determined for the first time, as shown in Figure 5. A total of 12 three-phase regions and 12 two-phase regions were measured. In this isothermal section, there are four ternary compounds, among which, τ1, τ3 and τ4 have solid solubility intervals, and seven binary compounds, three of which have solid solubility, which are Fe_2_Zr, FeZr_3_, Fe_2_Y.

**Figure 6:**
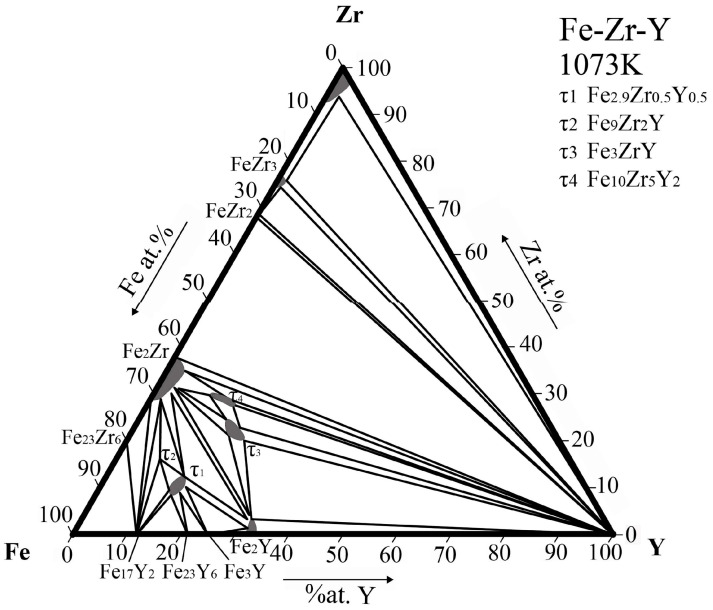
Isothermal section of the Fe-Zr-Y ternary system at 1073 K obtained in this work.

The isothermal section of Fe-Zr-Y ternary system at 1073 K is similar to the system at 973 K except that the maximum solid solubility of compounds τ1 and τ3 are slightly higher at 1073 K. Additionally, FeZr_2_ as a high temperature phase appeared at 1073 K. This is consistent with the binary optimized phase diagram, and therefore its appearance is reasonable and in accordance with expectation [30]. The 1073 K isothermal section obtained in this work is shown in Figure 6.

## 4. Discussion

The experimental data obtained from SEM, EPMA, XRD and EBSD examination were analyzed to determine the isothermal section and the phase relationship of the Fe-Zr-Y ternary system at 973 K and 1073 K. The phase relations in several key alloys were discussed in detail as follows.

### 4.1. Phase Equilibria at 973 K

A total of 24 alloy samples were prepared for the study of the phase equilibria of the Fe-Zr-Y ternary system at 973 K. The constituent phases of each alloy sample were summarized in Table 2.

**Figure 7:**
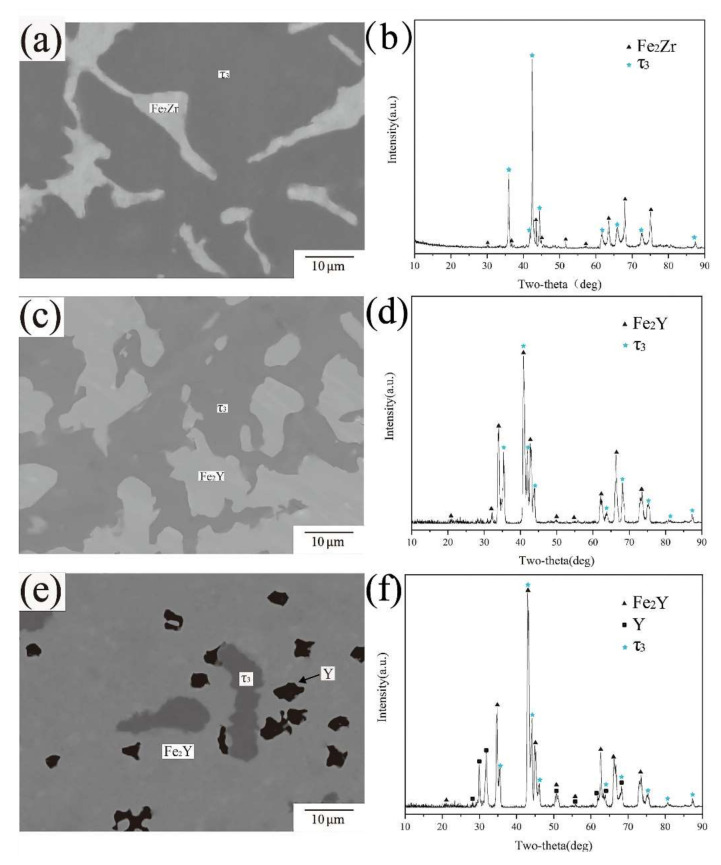
(**a**) BSE image of A13 (**b**) XRD pattern of A13 (**c**) BSE image of A1 of A24 (**d**) XRD pattern of A24 (**e**) BSE image of A7 (**f**) XRD pattern of A7.

BSE images and XRD patterns of alloy samples A7, A13 and A24 are shown in Figure 7a,c,e). EPMA-WDS results show that the dark gray phases in A7, A13 and A24 have the same composition, close to Fe60Zr20Y20, so they are confirmed as the same phase. However, this composition is different from the existing ternary compounds in the Fe-Zr-Y system. For the further identification of this phase, XRD analysis was performed on all three samples. In the XRD patterns of the three alloys, there are unrecorded characteristic peaks, the positions of which are basically fixed. Only a few peaks have slight displacement, which may be due to the change in lattice parameters. According to the above analysis, the ternary compound was identified as a new phase with the chemical formula Fe_3_ZrY and named τ3. Since no single phase of τ3 was obtained in this work, follow-up experiments are needed to explore the crystal structure of τ3. 

**Figure 8:**
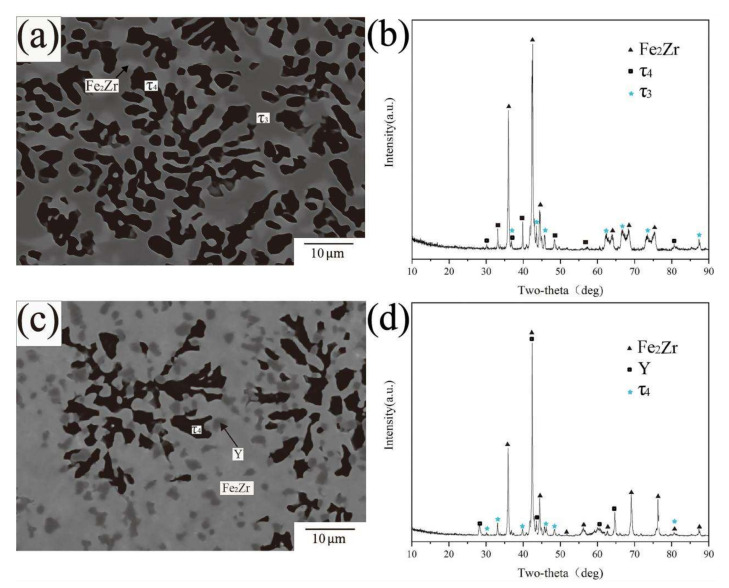
(**a**) BSE image of A2 (**b**) XRD pattern of A2 (**c**) BSE image of A9 (**d**) XRD pattern of A9.

There are three phases in the BSE images of alloy samples A2 and A9 (Figure 8a,c). According to the results of XRD and EPMA-WDS tests, all the phases are already identified except for the black phase. The compositions of the black phase in Figure 8a,c are Fe59.7Zr29.6Y10.5 and Fe56.2Zr27.9Y15.9, respectively. The stoichiometric ratios represent some differences between the two compositions, so the black phase was initially considered to be two different compounds. However, the XRD patterns (Figure 8b,d) show that the characteristic peaks of the two matched up, indicating that the crystal structures of the two phases were consistent, thus the two phases were confirmed to be the same compound. No ternary compound with similar stoichiometric ratio and diffraction peak matching was found in PDF cards. According to the results described above, the black phase was defined as the new ternary compound Fe_10_Zr_5_Y_2_ and named as τ4.

**Figure 9:**
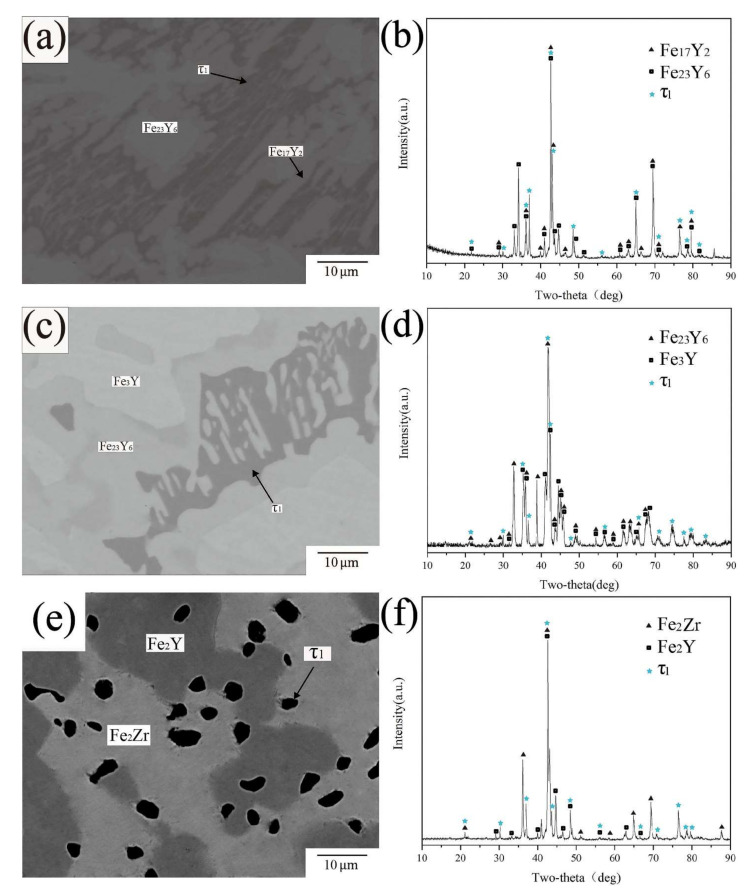
(**a**) BSE image of A5 (**b**) XRD pattern of A5 (**c**) BSE image of A17 (**d**) XRD pattern of A17 (**e**) BSE image of A10 (**f**) XRD pattern of A10.

The BSE images of alloy samples A5, A10 and A17 are shown in Figure 9a,c,e. The black phase in A10 and the dark gray phase in A5 and A17 have the same atomic ratio of Fe_2.9_Zr_0.5_Y_0.5_, which was denoted as τ1 phase. The XRD (as shown in Figure 9b,d,f) characteristic peaks of compound τ1 have fixed positions, which are different from all the existing PDF cards. According to the reported literature [41], Fe_2.9_Zr_0.5_Y_0.5_ was considered to be transformed from Fe_3_Y, as Zr atoms replaced Y atoms in Fe_3_Y. In the study of Itoh et al. [41], Fe_3_Y did not exist in the Fe-Zr-Y system, as it was considered to be present in the form of Fe_2.9_Zr_x_Y_1−x_ (0 ≤ x ≤ 1), meaning that Y would be replaced by Zr until Y disappears completely. This conclusion was not adopted in this work due to the following reasons: (1) Fe_2.9_Zr_0.5_Y_0.5_ would be adjacent to Fe_3_Y phase according to the reported theory [41], which is not consistent with what is shown in Figure 9a,c,e. Although there is Fe_3_Y shown in Figure 9c, it is separated by Fe_23_Y_6_, and τ1 is completely wrapped by Fe_23_Y_6_ instead of Fe_3_Y. (2) If Fe_2.9_Zr_x_Y_1−x_ (0 ≤ x ≤1) phase exists, the solubility range of Zr in Fe_3_Y would pass through most of the phase equilibrium region, which violates the law of phase equilibrium relation. In conclusion, the phase Fe_2.9_Zr_x_Y_1−x_ (0 ≤ x ≤ 1) is not adopted in this work.

**Figure 10:**
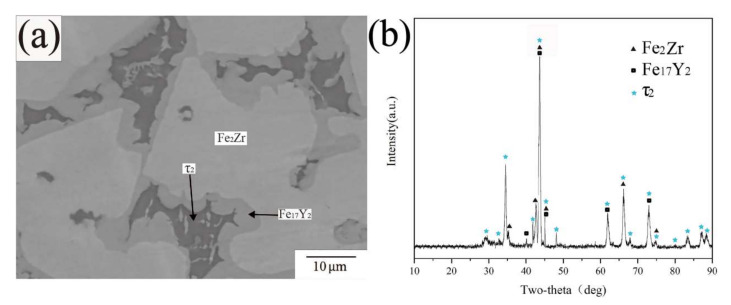
(**a**) BSE image of A4 (**b**) XRD pattern of A4.

BSE image Figure 10a illustrates the phase composition in alloy A4, which is composed of three phases. The results of EPMA-WDS show the continuously distributed matrix phase and the gray phase had the composition of Fe68.1Zr29.3Y2.6 and Fe88.2Y11.8, and were, respectively, determined as Fe_2_Zr and Fe_17_Y_2_ according to the XRD results of Figure 10b. The black phase labeled τ2 was presumed to be an unknown ternary compound at first, because the diffraction peaks cannot be matched by any PDF card corresponding to the crystal structure of the stable solid phase in the ternary system. However, they are indexed by a ThMn_12_ type crystal structure and obtain the characteristic peaks of Fe_9_Zr_2_Y, which coincide with τ2 in the literature [40]. Therefore, τ2 phase was finally identified as the ternary compound Fe_9_Zr_2_Y.

**Figure 11:**
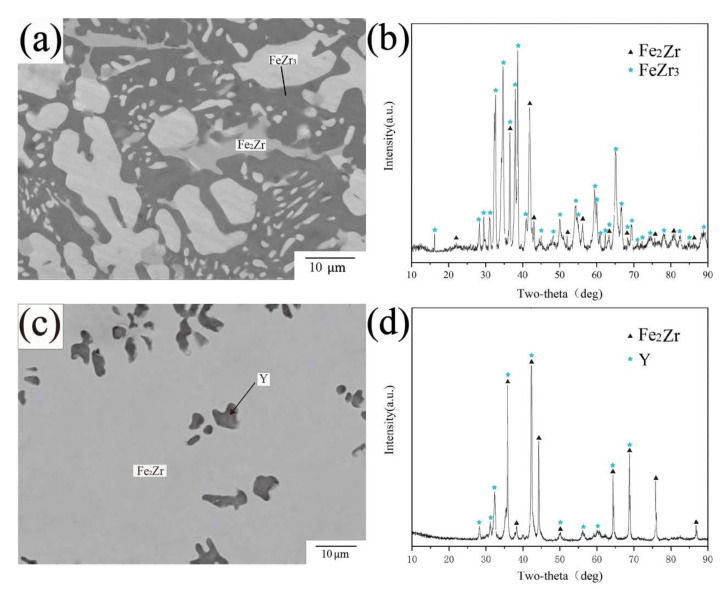
(**a**) BSE image of A16 (**b**) XRD pattern of A16 (**c**) BSE image of A18 (**d**) XRD pattern of A18.

Figure 11 shows the BSE images and XRD patterns of A16 and A18. According to the data from EPMA-WDS, the maximum atomic percentage of Zr in Fe_2_Zr is 37.6 at 973 K in A16. Combining Figure 11c with Figure 5, it can be seen that Y tends to precipitate in the form of metal simple substance most of the time, which is in accord with the results obtained by Nouri et al. [38] Meanwhile, Y phase is dispersed, indented and porous, which can be mainly ascribed to the result of rapid galvanic corrosion of the (Y), which acts as anode with Continuous-distributed matrix as cathode during polishing (or exposure to the moisture) [38].

### 4.2. Phase Equilibria at 1073 K

The phase equilibrium of the Fe-Zr-Y ternary system at 1073 K was also investigated in this work. A total of 25 alloy samples were annealed at 1073 K for 60 days. The phases and compositions of each alloy are listed in Table 3.

**Figure 12:**
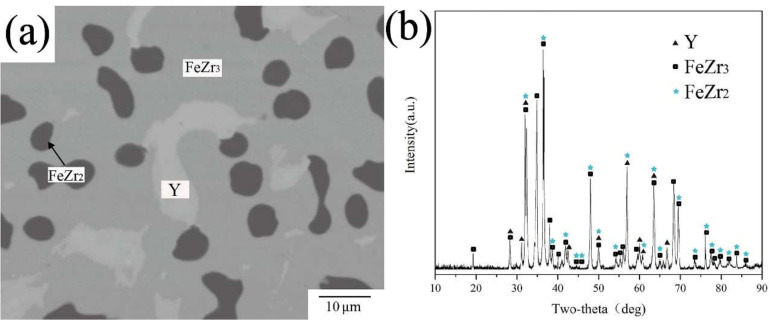
(**a**) BSE image of B23 (**b**) XRD pattern of B23.

With the increase in temperature, the solid solubility region of τ1 and Fe_2_Zr becomes larger, and a new high temperature phase FeZr_2_ appears. This result is consistent with the binary phase diagram of Fe-Zr, but the exact formation temperature of FeZr_2_ had not been reported to date. The XRD pattern of B23 in Figure 12b shows the same characteristic peaks of FeZr_2_ as standard PDF cards. Combined with EPMA-WDS data, it can be seen that the dark phase in BSE image Figure 12a is the binary compound FeZr_2_.

## 5. Conclusions

In this work, the phase relationship of the Fe-Zr-Y ternary system at 973 K and 1073 K combined with EPMA-EDS, XRD and SEM results was studied systematically. There are twelve and thirteen three-phase regions measured at 973 K and 1073 K, respectively. At 1073 K, the solution ranges of τ1-Fe_2.9_Zr_0.5_Y_0.5_ are from Fe73Zr12Y14 to Fe77Zr9Y13. The maximum solid solubility of Y in Fe_2_Zr is 4 at.% and atomic ratio of Fe_2_Zr is from Fe62Zr38 to Fe71Zr29 at 1073 K. New ternary compounds τ3-Fe_3_ZrY and τ4-Fe_10_Zr_5_Y_2_ were investigated. Those isothermal sections at 1073 K and 973 K provide the possibility of providing a thermodynamic description of the system through the CALPHAD method, allowing more reliable extrapolations of the Fe-Zr-Y system to be used in the nuclear industry.

## Figures and Tables

**Table 1:** Experimental and literature data on crystal structures and lattice parameters of the solid phases in the Fe-Zr-Y system.

Phase	Phase Prototype	Space Group		Lattice Parameters (nm)		Reference
			a	b	c	
α (Fe)	W	Im3¯m	0.29315	/	/	[42]
δ (Fe)	W	Im3¯m	0.29315	/	/	[42]
β (Zr)	W	Im3¯m	0.3568	/	/	[42]
γ (Fe)	Cu	$\mathrm{Fm}\bar{3}$m	0.36599	/	/	[42]
α (Zr)	Mg	P63/mmc	0.323178	/	0.514831	[28]
Fe_2_Zr_C15	MgCu_2_	$\mathrm{Fd}\bar{3}$m	0.702	/	/	[28]
Fe_23_Zr_6_	Mn_23_Th_6_	$\mathrm{Fm}\bar{3}$m	1.172	/	/	[28]
FeZr_2_	Al2Cu	I4/mcm	0.638	/	0.56	[28]
FeZr_3_	BRe_3_	Cmcm	0.332	1.1	0.882	[28]
Fe_17_Y_2_	Th_2_Zn_17_	$R\bar{3}$m	0.846	/	1.241	[43]
Fe_23_Y_6_	Th6Mn_23_	$\mathrm{Fm}\bar{3}$m	1.2082	/	/	[43]
Fe_3_Y	PuNi_3_	$R\bar{3}$m	0.515	/	2.46	[43]
Fe_2_Y	MgCu_2_	$\mathrm{Fd}\bar{3}$m	7.363	/	/	[43]
Fe_9_Zr_2_Y	ThMn_12_	I4/mmm	0.8662	0.8662	0.50226	[40]
Fe_2.9_Zr_0.5_Y_0.5_	/	/	0.5067	0.8634	2.451	[41]

**Table 2:** Constituent phases and compositions in the annealed Fe-Zr-Y alloys at 973 K for 90 days.

Alloy (No.)	Chemical Composition	Nominal Composition (at.%)			Experimental Results (at.%)			Phase Determination
		Fe	Zr	Y	Fe	Zr	Y	
A1	Fe10Zr70Y20	10	70	20	62.61	37.23	0.16	Fe_2_Zr
					24.35	74.42	1.23	FeZr_3_
					0.29	0.13	99.58	Y
A2	Fe60Zr30Y10	60	30	10	59.22	24.46	16.32	τ3
					59.77	29.61	10.62	τ4
					65.85	31.84	2.31	Fe_2_Zr
A3	Fe10Zr60Y30	10	60	30	23.31	75.16	1.53	FeZr_3_
					2.81	95.82	1.37	Zr
					98.87	0.15	0.98	Y
A4	Fe77Zr16Y7	77	16	7	68.14	29.31	2.55	Fe_2_Zr
					75.81	16.12	8.07	τ2
					88.24	0.21	11.55	Fe_17_Y_2_
A5	Fe80Zr4Y16	80	4	16	75.49	10.12	14.39	τ1
					78.74	0.28	20.98	Fe_23_Y_6_
					88.26	0.17	11.57	Fe_17_Y_2_
A6	Fe45Zr20Y35	45	20	35	57.34	23.89	18.77	τ3
					56.54	27.75	15.71	τ4
					0.21	0.16	99.63	Y
A7	Fe50Zr10Y40	50	10	40	59.14	21.3	19.56	τ3
					99.54	0.16	0.30	Y
					65.35	3.34	31.31	Fe_2_Y
A8	Fe65Zr20Y15	65	20	15	65.86	31.84	2.30	Fe_2_Zr
					61.11	22.54	16.35	τ3
					65.38	3.36	31.26	Fe_2_Y
A9	Fe55Zr30Y15	55	30	15	62.51	35.48	2.01	Fe_2_Zr
					57.17	29.6	13.23	τ4
					0.11	0.25	99.64	Y
A10	Fe70Zr15Y15	70	15	15	74.62	11.77	13.61	τ1
					65.34	2.74	31.92	Fe_2_Y
					66.54	30.86	2.60	Fe_2_Zr
A11	Fe62Zr32Y6	62	32	6	65.13	33.11	1.76	Fe_2_Zr
					59.35	30.36	10.29	τ4
A12	Fe20Zr60Y20	20	60	20	0.15	0.24	99.61	Y
					23.59	74.42	1.99	FeZr_3_
A13	Fe61Zr25Y14	61	25	14	59.44	24.42	16.14	τ3
					65.87	31.82	2.31	Fe_2_Zr
A14	FeZr74Y25	1	74	25	1.52	96.93	1.55	Zr
					0.14	0.18	99.68	Y
A15	Fe15Zr84Y	15	84	1	1.19	95.92	2.89	Zr
					23.21	75.96	0.83	FeZr_3_
A16	Fe29Zr70Y	29	70	1	24.33	74.41	1.26	FeZr_3_
					62.45	37.44	0.11	Fe_2_Zr
A17	Fe75Zr6Y19	75	6	19	74.71	9.69	15.6	τ1
					75.33	0.25	24.42	Fe_3_Y
					78.75	0.33	20.92	Fe_23_Y_6_
A18	Fe55Zr30Y15	55	30	15	62.31	35.87	1.82	Fe_2_Zr
					0.34	0.13	99.53	Y
A19	Fe50Zr25Y25	50	25	25	56.25	27.91	15.84	τ4
					0.21	0.11	99.68	Y
A20	Fe39ZrY60	39	1	60	65.45	1.86	32.69	Fe_2_Y
					0.24	0.23	99.53	Y
A21	Fe70ZrY29	70	1	29	66.87	0.16	32.97	Fe_2_Y
					73.41	0.54	26.05	Fe_3_Y
A22	Fe66Zr10Y24	66	10	24	65.66	2.74	31.6	Fe_2_Y
					66.35	30.82	2.83	Fe_2_Zr
A23	Fe4Zr8Y88	4	8	88	80.33	19.37	0.30	Fe_23_Zr_6_
					88.21	0.15	11.64	Fe_17_Y_2_
					98.56	0.74	0.70	Fe
A24	Fe60Zr18Y22	60	18	22	65.39	3.18	31.43	Fe_2_Y
					60.25	21.24	18.51	τ3

**Table 3:** Constituent phases and compositions in the annealed Fe-Zr-Y alloys at 1073 K for 60 days.

Alloy (No.)	Chemical Composition	Nominal Composition (at.%)			Experimental Results (at.%)			Phase Determination
		Fe	Zr	Y	Fe	Zr	Y	
B1	Fe10Zr70Y20	10	70	20	62.21	37.72	0.07	Fe_2_Zr
					31.72	67.37	0.91	FeZr_2_
					0.13	0.21	99.66	Y
B2	Fe60Zr30Y10	60	30	10	59.25	24.41	16.34	τ3
					59.73	29.66	10.61	τ4
					65.42	31.21	3.37	Fe_2_Zr
B3	Fe10Zr60Y30	10	60	30	24.34	74.48	1.18	FeZr_3_
					93.86	3.84	2.3	Zr
					0.35	0.13	99.52	Y
B4	Fe77Zr16Y7	77	16	7	68.15	29.33	2.52	Fe_2_Zr
					75.84	16.17	7.99	τ2
					88.25	0.16	11.59	Fe_17_Y_2_
B5	Fe80Zr4Y16	80	4	16	77.48	9.59	12.93	τ1
					78.77	0.27	20.96	Fe_23_Y_6_
					88.22	0.15	11.63	Fe_17_Y_2_
B6	Fe45Zr20Y35	45	20	35	57.63	22.74	19.63	τ3
					56.54	27.70	15.76	τ4
					0.12	0.25	99.63	Y
B7	Fe50Zr10Y40	50	10	40	58.29	20.01	21.7	τ3
					0.35	0.16	99.49	Y
					65.31	3.36	31.33	Fe_2_Y
B8	Fe65Zr20Y15	65	20	15	65.47	31.28	3.25	Fe_2_Zr
					60.55	21.16	18.29	τ3
					65.34	12.61	22.05	Fe_2_Y
B9	Fe55Zr30Y15	55	30	15	61.89	34.82	3.29	Fe_2_Zr
					56.68	28.8	14.52	τ4
					0.51	0.20	99.29	Y
B10	Fe70Zr15Y15	70	15	15	73.14	12.48	14.38	τ1
					65.33	2.71	31.96	Fe_2_Y
					66.59	30.65	2.76	Fe_2_Zr
B11	Fe62Zr32Y6	62	32	6	63.23	32.45	4.32	Fe_2_Zr
					10.28	30.79	58.93	τ4
B12	Fe20Zr60Y20	20	60	20	0.41	0.38	99.21	Y
					1.73	75.2	23.07	FeZr_3_
B13	Fe61Zr25Y14	61	25	14	60.17	23.12	16.71	τ3
					65.54	31.35	3.11	Fe_2_Zr
B14	FeZr74Y25	1	74	25	1.81	96.55	1.64	Zr
					0.15	0.25	99.6	Y
B15	Fe15Zr84Y	15	84	1	0.87	94.83	4.3	Zr
					22.61	76.52	0.87	FeZr_3_
B16	Fe29Zr70Y	29	70	1	24.45	74.89	0.66	FeZr_3_
					31.31	68.61	0.08	FeZr_2_
B17	Fe75Zr6Y19	75	6	19	75.74	9.57	14.69	τ1
					75.31	0.36	24.33	Fe_3_Y
					77.51	0.85	21.64	Fe_23_Y_6_
B18	Fe55Zr30Y15	55	30	15	61.94	36.13	1.93	Fe_2_Zr
					0.10	0.21	99.69	Y
B19	Fe50Zr25Y25	50	25	25	56.24	28.29	15.47	τ4
					0.32	0.15	99.53	Y
B20	Fe39ZrY60	39	1	60	65.41	1.81	32.78	Fe_2_Y
					0.14	0.32	99.54	Y
B21	Fe70ZrY29	70	1	29	66.96	32.52	0.52	Fe_2_Y
					75.06	0.10	24.84	Fe_3_Y
B22	Fe66Zr10Y24	66	10	24	65.54	3.36	31.1	Fe_2_Y
					66.11	30.91	2.98	Fe_2_Zr
B23	Fe20Zr60Y20	20	60	20	22.95	75.47	1.58	FeZr_3_
					0.13	0.13	99.74	Y
					31.20	68.64	0.16	FeZr_2_
B24	Fe84.5Zr12.5Y3	84.5	12.5	3	80.31	19.61	0.08	Fe_23_Zr_6_
					88.87	0.83	10.3	Fe_17_Y_2_
					99.15	0.17	0.68	Fe
B25	Fe60Zr18Y22	60	18	22	65.32	3.37	31.31	Fe_2_Y
					59.21	20.29	20.5	τ3

## Data Availability

Not applicable.
